# The pathophysiological link between type 1 diabetes and MASLD: insights into insulin resistance and liver dysfunction

**DOI:** 10.1007/s40618-025-02621-5

**Published:** 2025-06-27

**Authors:** José L. Herrera-Ojeda, Ray S. Blanco-Palma, Norberto C. Chávez-Tapia, Misael Uribe, Eduardo E. Montalvo-Javé, Natalia Nuño-Lámbarri

**Affiliations:** 1 Traslational Research Unit, Medica Sur Clinic & Foundation, Mexico City, Mexico; 2 Department of Surgery Faculty of Medicine, The National Autonomous University of Mexico (UNAM), Mexico City, Mexico; 3 Obesity and Digestive Diseases Unit, Medica Sur Clinic & Foundation, Mexico City, Mexico

**Keywords:** Glucose, Insulin, Insulin resistance, Liver disease, Steatohepatitis

## Abstract

Type 1 Diabetes Mellitus (T1DM) is an autoimmune disorder characterized by the destruction of pancreatic β-cells, leading to significant endogenous insulin deficiency. In this context, inflammation plays a crucial role in the pathogenesis of the disease. Traditionally, insulin resistance has been associated with Type 2 Diabetes Mellitus (T2DM); however, recent studies have shown that it also occurs in a significant proportion of T1DM patients. Regarding the prevalence of Non-Alcoholic Fatty Liver Disease associated with metabolic dysfunction (MASLD), variations are observed depending on the studied population and diagnostic method used, although there has been a global increase in this condition in T1DM patients. MASLD is closely linked to insulin resistance, both hepatic and peripheral, suggesting that MASLD progression is associated with worsening insulin resistance. The relationship between both pathologies is bidirectional, as the presence of one can accelerate the progression of the other. When both coexist, the natural history of both diseases is altered, increasing the risk of complications and worsening patient prognosis.

## Introduction

Type 1 Diabetes Mellitus (T1DM) is an autoimmune disorder characterized by the progressive destruction of pancreatic β-cells, leading to significant endogenous insulin deficiency. The global prevalence of T1DM is estimated at 95 cases per 100,000 people. Worldwide, the incidence of T1DM is increasing, particularly among children, although it is estimated that between 25 and 50% of new diagnoses occur in adults. This disease is associated with genetic alterations triggered by environmental factors, leading to a slow progression that may span from months to years. This explains why, although the incidence peaks during puberty, T1DM can present at any age, and patients can live many decades after diagnosis. Therefore, the prevalence of T1DM is higher in adults than in children. [1, 2].

The prolonged latency period in T1DM reflects the substantial number of functional β-cells that must be lost before hyperglycemia develops. Unlike Type 2 Diabetes Mellitus (T2DM), where the reduction in insulin secretion is primarily associated with factors like insulin resistance, T1DM is characterized by an autoimmune destruction of β-cells. [3] However, inflammation plays a common role in both types of diabetes, contributing to β-cell dysfunction in the pancreatic islets. Studies using the hyperinsulinemic-euglycemic clamp technique have shown that T1DM patients exhibit greater insulin resistance compared to healthy individuals, which increases their risk of developing metabolic and cardiovascular complications associated with insulin resistance. [4].

Metabolic Dysfunction-Associated Steatotic Liver Disease (MASLD), formerly known as Metabolic Dysfunction-Associated Fatty Liver Disease (MAFLD) and Non-Alcoholic Fatty Liver Disease (NAFLD), is characterized by the accumulation of fat in more than 5% of hepatocytes, detected by imaging or biopsy. The condition is associated with at least one of the following five cardiometabolic criteria:

1. Body mass index (BMI) ≥ 23 kg/m^2^ in Asians or waist circumference ≥ 94 cm in men and ≥ 80 cm in women.
2. Fasting serum glucose ≥ 100 mg/dl, Glycated hemoglobin (HbA1c) ≥ 5.7%, or a diagnosis/treatment of Type 2 Diabetes.
3. Blood pressure ≥ 130/85 mmHg or specific antihypertensive treatment.
4. Plasma triglycerides ≥ 150 mg/dl or lipid-lowering treatment.
5. High-density lipoprotein (HDL) ≤ 40 mg/dl in men and ≤ 50 mg/dl in women or lipid-lowering treatment.

This redefinition addresses the limitations of the NAFLD term, which was based on exclusion criteria and did not adequately reflect the causal relationship between metabolic dysfunction and liver disease. [5–7].

MASLD is considered the hepatic manifestation of metabolic syndrome due to its association with various factors beyond diabetes mellitus and obesity. While diabetes mellitus plays a key role in the progression of MASLD towards metabolic dysfunction-associated steatohepatitis (MASH), insulin resistance is recognized as the most important risk factor for MASLD development, even more so than obesity. [8] (Table 1).

**Table 1:** Comparison of the main characteristics of the NAFLD, MAFLD and MASLD nomenclatures

Features	NAFLD	MAFLD	MASLD
Definition	Hepatic steatosis > 5% according to imaging or histological studies, in the absence of excessive alcohol consumption, steatogenic drugs and hereditary disorders Defined by negative criteria	Hepatic steatosis > 5% detected by imaging, biomarker or histological studies. With one of the following: - Overweight/obesity - Diabetes Mellitus 2 - Two or more metabolic criteria Defined by positive criteria	Hepatic steatosis > 5% diagnosed by imaging or biopsy, in the presence of at least one or more cardiometabolic risk factors
Relationship with alcohol	Patients with significant alcohol consumption were excluded	It does not exclude patients with alcohol consumption but does not suggest any threshold for subcategorization	Includes overlap with significant alcohol consumption under the MetALD subcategory
Relationship with metabolic syndrome	It does not clearly establish a causal association between metabolic dysfunction and liver disease	Emphasizes the central role of metabolic dysfunction in the etiology of hepatic steatosis	It integrates cardiometabolic factors in its definition, highlighting their role in hepatic fat accumulation
Limitations	Does not reflect underlying Pathophysiology, diagnosis based on exclusion; potentially stigmatizing nomenclature	It retains the term"fatty", which can be pejorative; it does not adequately address the interaction with alcohol	It has limited supporting evidence because it is a recently suggested term
Conceptual evolution	Proposed in the 1990 s rapidly adopted globally due to the lack of a more appropriate terminology	Proposed in 2020 by Eslam et al., with growing acceptance in countries with a high prevalence of metabolic syndrome, although with some criticism	Adopted in 2023 by international hepatological associations after a modified Delphi consensus as an improvement over NAFLD and MAFLD

While the association between MASLD and T2DM has been extensively characterized, MASLD in the context of T1DM represents a distinct clinical and biological entity that warrants dedicated attention. Unlike T2DM, where MASLD develops in the presence of endogenous hyperinsulinemia and insulin resistance, T1DM is defined by autoimmune β-cell destruction and absolute insulin deficiency. However, a growing subset of T1DM patients develops insulin resistance, often due to intensive insulin therapy, weight gain, or genetic predisposition. This overlapping phenotype is referred to as “double diabetes,” describing individuals with T1DM who simultaneously exhibit clinical features of T2DM, such as obesity, insulin resistance, and metabolic syndrome. This dual pathology amplifies the risk of hepatic steatosis through unique mechanisms, including altered insulin gradients (portal vs. systemic), peripheral hyperinsulinemia, and ectopic lipid deposition. Consequently, MASLD in T1DM follows a different pathophysiological trajectory and may lead to accelerated progression toward steatohepatitis, fibrosis, and cardiovascular complications. Recognizing this overlap is essential for tailored diagnostic strategies and therapeutic interventions. [9, 10].

## MASLD and T1DM

MASLD is the leading cause of chronic liver disease worldwide, with a prevalence ranging from 15 to 70%, depending on factors such as ethnic variation and diagnostic methods. [11].

Regarding the prevalence of MASLD in patients with T1DM, studies employing imaging techniques and serum liver enzyme measurements report varying results. The considerable heterogeneity in MASLD prevalence among individuals with T1DM reported across studies can be attributed to several methodological and demographic factors. First, age is a critical determinant; pediatric and adolescent populations [10, 12, 13, 16] generally show lower MASLD prevalence compared to adult cohorts [14, 15], likely due to shorter diabetes duration and lower cumulative metabolic burden. Second, duration of T1DM is not uniformly reported but has been linked to progressive insulin resistance and hepatic steatosis, potentially explaining higher rates in older populations. Third, ethnic background may influence hepatic fat accumulation and insulin sensitivity; for instance, the higher prevalence observed in Egyptian youth [16] may reflect both genetic susceptibility and regional metabolic risk profiles. Finally, diagnostic methodology significantly impacts prevalence estimates. Imaging techniques such as ultrasound are operator-dependent and less sensitive than MRI or histology, whereas reliance on serum aminotransferase levels may underestimate steatosis in asymptomatic patients. These differences underscore the need for standardized diagnostic criteria and cohort characterization to improve the comparability and interpretability of MASLD prevalence data in T1DM populations. (Table 2).

**Table 2:** MASLD in T1DM: prevalence summary

Sample size	Age range	Diagnostic method	MASLD prevalence	Key confounders	Study
106	8 mo– 15 y	Ultrasound	11.3	BMI, Age	(12)
110	8–17 y	Ultrasound	15.5	HbA1c, BMI	(13)
301	Adults	Liver Enzymes & Imaging	36.8	BMI, Duration of T1DM	(14)
220	31–49 y	Liver Enzymes	25.9	Vitamin D, BMI	(15)
244	Children & Adolescents	Ultrasound	27.5	HbA1c, Glycemic Control	(10)
74	8–18 y	Ultrasound	62.2	BMI, Duration of T1DM, Glycemic Control	(16)

These factors play a crucial role in interpreting the differences in epidemiological data, highlighting the need to standardize diagnostic and evaluation methods to improve the understanding of this condition in T1DM patients. Given the global increase in MASLD prevalence in this patient group, it is essential to implement more uniform and accurate diagnostic strategies to enhance our understanding of this pathology.

## Dysfunction of insulin in T1DM and MASLD

The physiology of insulin secretion is biphasic. The first phase consists of a rapid peak prior to glucose stimulation, followed by a decline to near-basal levels. The second phase involves a gradual increase in insulin levels until reaching a maximum that persists during glucose exposure. Insulin interacts with a membrane receptor composed of four subunits: two α and two β. The α subunit is extracellular and facilitates insulin binding to the receptor, while the β subunit contains extracellular, transmembrane, and intracellular domains. Binding of insulin to the α subunit induces a conformational change in the β subunit, which activates receptor autophosphorylation and initiates intracellular signaling cascades. [17].

Once the tyrosine kinase of the β subunit is activated, it promotes the phosphorylation of insulin receptor substrate (IRS) at its serine, tyrosine, and threonine residues. There are two main IRS isoforms—IRS-1 and IRS-2—expressed in hepatocytes. [18] Phosphorylated IRS associates with phosphatidylinositol-3-kinase (PI3K), a molecule implicated in cellular growth through the activation of S6 kinase (S6K) and cellular proliferation via the activation of protein kinase B (PKB) in conjunction with 3'-Phosphoinositide-dependent protein kinase 1 (PDK1). Furthermore, PI3K has been shown to mediate the translocation of glucose transporter (GLUT) transporters from intracellular vesicles to the plasma membrane, thereby facilitating glucose uptake. [19, 20].

In the liver, insulin exerts its effects by suppressing glycogenolysis and gluconeogenesis while promoting glycogen synthesis and lipogenesis through distinct signaling pathways. Insulin stimulates glycogen synthesis via the PI3K cascade, leading to AKT phosphorylation, which inactivates glycogen synthase kinase-3 (GSK-3). The inhibition of gluconeogenesis is mediated by AKT-dependent phosphorylation and nuclear exclusion of FOXO1, which suppresses the expression of glucose-6-phosphatase (G6Pase) and phosphoenolpyruvate carboxykinase (PEPCK). [21].

Conversely, lipogenesis is stimulated by hyperinsulinemia through increased concentrations of the sterol regulatory element-binding protein (SREBP-1c), which promotes the expression of hepatic lipogenic genes, such as liver-type pyruvate kinase (LPK), fatty acid synthase (FAS), and acetyl-CoA carboxylase (ACC). Hyperglycemia, on the other hand, also drives lipogenesis by activating the carbohydrate response element-binding protein (ChREBP). [22].

Insulin stimulates glycogen synthesis and suppresses gluconeogenesis via the PI3K-AKT pathway, a mechanism well-characterized in both T1DM and T2DM models. Lipogenesis is also promoted via SREBP-1c and ChREBP activation, largely established in T2DM and MASLD, and more recently supported in T1DM through indirect evidence of hepatic lipid accumulation.

**Fig. 1:**
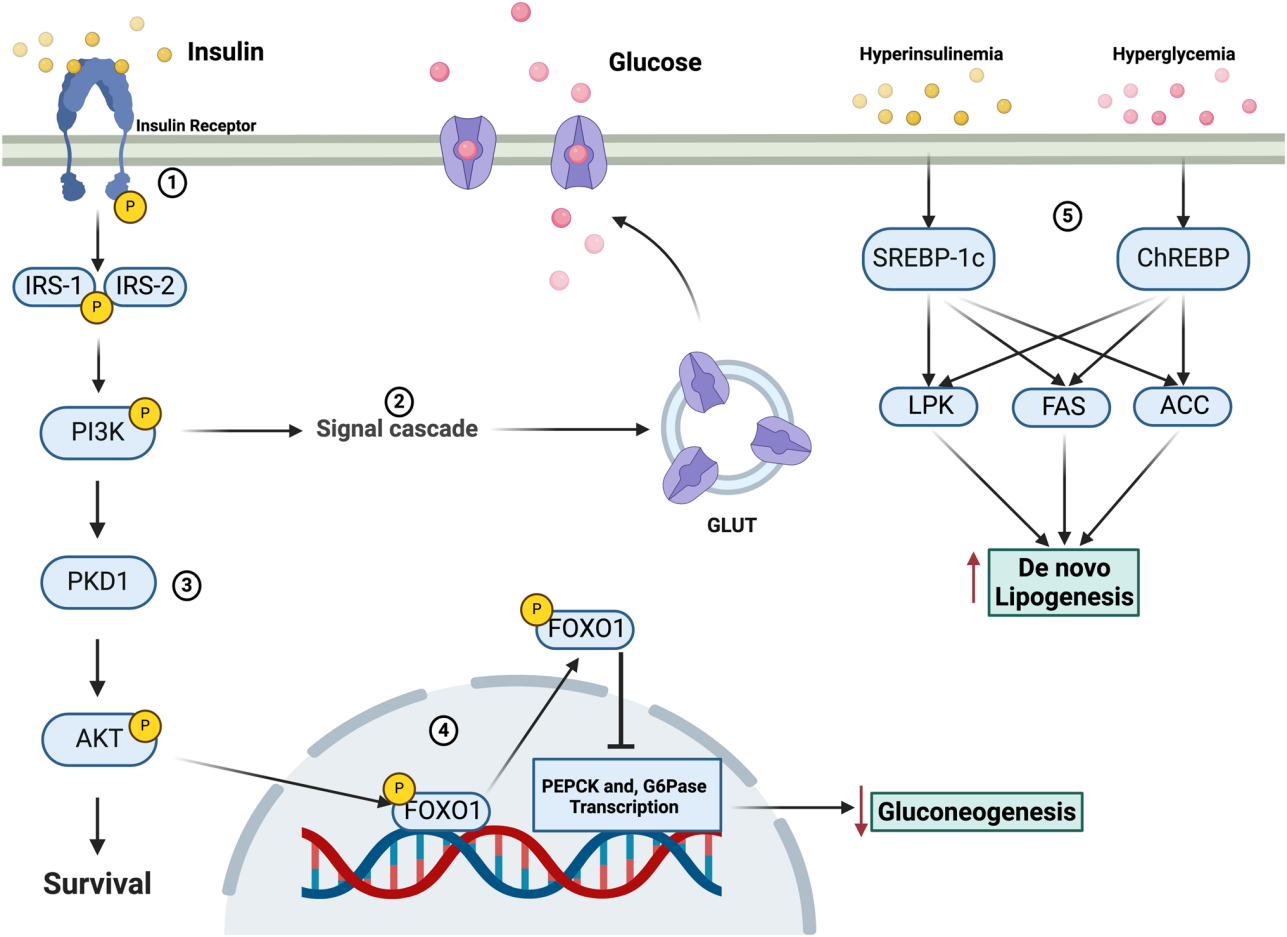
The action of insulin in the liver. 1) Insulin binds to its receptor, which stimulates its autophosphorylation; once activated, it promotes IRS-1 and IRS-2 phosphorylation that activate PI3K. 2) The PI3K signaling pathway causes GLUT exocytosis and translocation, allowing glucose internalization. 3) On the other hand, the interaction of PI3K with PDK1 activates AKT, which promotes cell proliferation and survival. 4) AKT-dependent phosphorylation excludes FOXO1 from the nucleus suppressing the expression of PEPCK and G6Pase, decreasing gluconeogenesis. 5) On the other hand, hyperinsulinemia and hyperglycemia increase the concentration of SREBP-1c and ChREBP, which stimulate the expression of hepatic lipogenic genes such as LPK, FAS, and ACC, leading to an increase in de novo lipogenesis

Recent studies suggesting that IRS-1 primarily regulates lipogenesis, while IRS-2 mediates gluconeogenesis suppression remain largely speculative and are mainly derived from MASLD and animal models. [18, 23] (Fig. 1).

Insulin resistance has traditionally been associated with T2DM; however, its presence has been significantly demonstrated in patients with T1DM. The term “double diabetes” was first described by Teupe, who observed that T1DM patients with a family history of T2DM exhibited a higher propensity for overweight and required increased insulin doses to achieve optimal blood glucose levels. In some cases, these patients were unable to achieve desired glycemic targets despite escalating insulin doses.

Double diabetes can be defined as the coexistence of T1DM with clinical features characteristic of T2DM, resulting in exacerbated insulin resistance. The concept “double diabetes” is well-documented in T1DM cohorts and supported by population-based data linking intensive insulin therapy, obesity, and poor glycemic control to increased insulin resistance. [24] Having a first-degree relative with T2DM increases the risk of developing insulin resistance, serving as an independent predictor of weight gain and increasing the risk of coronary artery disease (CAD) with an odds ratio (OR) of 1.62 when one family member is affected and an OR of 5.13 when two family members are affected. One of the key contributors to insulin resistance in T1DM patients is intensive insulin therapy. [25].

The association between insulin resistance and overweight/obesity in T1DM is robust, and confirmed by longitudinal cohorts such as the European Diabetes Study (EURODIAB), patients receiving intensive insulin therapy and who gained more than 5 kg over 7.3 years showed increases in plasma triglycerides, total cholesterol, low-density lipoprotein (LDL) levels, as well as systolic and diastolic blood pressure, alongside decreases in plasma HDL levels and HbA1c. [26].

Another contributing factor to insulin resistance in T1DM is the high prevalence of overweight and obesity. For instance, a prevalence of overweight was reported in 22.1% of T1DM patients, compared to only 12.6% among lean individuals. [27, 28] After an 18-year follow-up, the prevalence of overweight increased to 47%, while obesity rose to 28.6%, highlighting the growing incidence of these conditions in a population traditionally regarded as lean. [29] Poor glycemic control also contributes to insulin resistance in T1DM. Patients with poor glycemic control (HbA1c 8.5 ± 0.4%) exhibit increased gluconeogenesis and higher fasting glucose levels compared to those with reasonable control or without T1DM. [30, 31].

Furthermore, MASLD has been observed in 15.5% of T1DM patients with poor glycemic control (HbA1c 9.91 ± 0.56), and even higher prevalence rates (HbA1c 13.14 ± 1.82) have been reported, independent of variables such as age and diabetes duration. [13].

A similar trend has been observed in pediatric patients with T1DM, where MASLD prevalence reached 21% and was linked to poor glycemic control, with a mean HbA1c of 12.14%. [10] In summary, the role of poor glycemic control in driving hepatic gluconeogenesis and MASLD progression is well established in T1DM patients, particularly through elevated HbA1c levels and associated hepatic steatosis.

Insulin resistance in T1DM has also been associated with the method of insulin administration. Subcutaneous exogenous insulin therapy may fail to replicate the physiological profile of endogenous insulin secretion. This imbalance leads to higher systemic insulin concentrations, promoting peripheral hyperinsulinemia, insulin resistance, and ectopic fat deposition in the liver. Additional factors, such as variability in insulin absorption, lipodystrophy at injection sites, and differences in therapeutic regimens, may further exacerbate metabolic dysfunction and contribute to the development of MASLD in T1DM patients. [10, 32].

Under physiological conditions, insulin concentrations in the portal vein are 2–4 times higher than those in the systemic circulation. However, in T1DM patients, the absence of endogenous insulin production results in a 1:1 ratio of portal to systemic insulin concentrations. This loss of the physiological gradient reduces peripheral insulin sensitivity while increasing hepatic insulin sensitivity. [33, 34] The impact of subcutaneous insulin therapy on portal/systemic gradients and subsequent hepatic steatosis, while biologically plausible, remains speculative and is primarily inferred from theoretical models and extrapolation from insulin pharmacokinetics**.**

Hepatic insulin resistance can be defined as impaired insulin-mediated suppression of glycogen breakdown and increased gluconeogenesis in the liver. Given the liver’s pivotal role in glucose and lipid metabolism regulation, its dysfunction exacerbates adipocyte dysfunction, creating a vicious cycle that worsens diabetes progression. Hepatic insulin resistance—characterized by impaired suppression of gluconeogenesis—is a well-recognized phenomenon in both T1DM and T2DM, although direct mechanistic studies in T1DM are fewer. [35].

The hyperinsulinemic-euglycemic clamp remains the gold standard for diagnosing insulin resistance in humans; however, its invasive and time-intensive nature limits its use in clinical practice. [36, 37] Insulin resistance, a hallmark of metabolic syndrome, is characterized by elevated blood pressure, central obesity, impaired glucose metabolism, increased triglycerides, and reduced HDL levels. [38] To facilitate the assessment of insulin sensitivity in daily clinical practice, the estimated glucose disposal rate (eGDR) formula was developed:

$$\begin{aligned}\text{eGDR} \:(\text{mg/kg/min}) & = 21,158 + (-0,09 \times \text{WC}) \\&+ (-3,407 \times \text{HTN}) + (-0,551 \times \text{HbA1c}). \end{aligned}$$

Where WC is waist circumference (cm), and HTN indicates hypertension (0 = no, 1 = yes). This formula has been validated for estimating insulin sensitivity in T1DM patients. [37] The importance of these variables lies in the positive correlation between waist-to-hip ratio, triglyceride levels, and blood pressure, and their negative correlation with HDL levels. Additionally, waist-to-hip ratio is associated with increased risk of metabolic complications in T1DM. [39] Lower eGDR values indicate greater insulin resistance.

Significant ethnic differences in eGDR values have been observed, with African American and Hispanic populations exhibiting the lowest values, emphasizing the importance of considering genetic and environmental factors in assessing and managing insulin resistance across diverse populations. [37, 40] The Ethnic differences in eGDR values and their implications on MASLD progression are still under investigation, and current data are mostly observational., and its correlation with cardiovascular and microvascular outcomes is increasingly validated. Also, ethnic differences in eGDR values and their implications on MASLD progression are still under investigation*,* and current data are mostly observational.

Insulin resistance, as evaluated by eGDR, is a critical factor for cardiovascular events in T1DM, surpassing traditional risk factors such as hypertension, overweight, or dyslipidemia. Studies have identified an eGDR value below 2.34 mg/kg/min as associated with a higher risk of cardiovascular events (AUC 78.5, p < 0.001), with a sensitivity of 95.5% and a specificity of 99.4%. [41] In another study, lower eGDR values were linked to microvascular complications, with diabetic retinopathy patients showing an eGDR of 5.97 ± 1.2 mg/kg/min, those with peripheral neuropathy 5.06 ± 0.4 mg/kg/min, and those with diabetic nephropathy 5.79 ± 1.5 mg/kg/min. An eGDR below 6 mg/kg/min doubles the risk of major cardiovascular events or mortality (HR 2.22, 95% CI 1.79–2.75). However, among patients with eGDR < 5.6 mg/kg/min, only 80% were classified as insulin-resistant when compared to the hyperinsulinemic-euglycemic clamp technique, underscoring the need for precise interpretation of this indicator. [36, 40].

In recent years, the phenomenon of selective insulin resistance has been described, characterized by differential alterations in insulin receptor signaling pathways. In this phenomenon, IRS-1 signaling remains active, promoting the expression of lipogenic enzymes such as FAS, whereas IRS-2 signaling, which inhibits gluconeogenic enzymes like PEPCK and G6Pase, is impaired. This imbalance contributes to the development of metabolic complications and the progression of hepatic and cardiovascular alterations in T1DM patients. [23] Selective insulin resistance involving IRS-1 preservation and IRS-2 inhibition is an emerging concept, largely based on human MASLD liver biopsy studies and remains speculative in the context of T1DM.

It is important to acknowledge that many of the mechanisms described in this section are supported primarily by observational studies or derived from animal models, with limited direct evidence in human cohorts specifically affected by both T1DM and MASLD. Consequently, several conclusions are based on extrapolations from T2DM or general MASLD populations. Additionally, while the eGDR is a useful surrogate marker for insulin sensitivity and has shown strong correlations with cardiovascular and microvascular outcomes in T1DM, its validation in large, imaging-confirmed MASLD cohorts within the T1DM population is lacking. Therefore, the use of eGDR in this context should be interpreted cautiously and complemented by additional diagnostic approaches when assessing metabolic risk.

## The dual impact of type 1 diabetes and MASLD

The presence of MASLD in patients with T1DM is associated with a higher prevalence of both microvascular and macrovascular complications. Key complications such as cerebrovascular disease, myocardial infarction, atherosclerosis, and peripheral vascular disease have been consistently observed in T1DM-MASLD populations, including asymptomatic cases identified via Doppler ultrasound. [42, 43].

Furthermore, individuals with T1DM and MASLD are at an increased risk of developing chronic kidney disease (CKD) due to impaired renal function. [44] A meta-analysis comprising 13 observational studies demonstrated that hepatic steatosis is associated with a > 1.5-fold increased long-term risk of developing stage ≥ 3 CKD, both in patients with and without T1DM. [45].

Cardiovascular disease (CVD) remains the leading cause of mortality in patients with MASLD, with a significantly higher prevalence and nearly double the incidence of cardiovascular events compared to individuals without this condition. Another meta-analysis reported that MASLD increases the risk of non-fatal cardiovascular events (RR 1.40; 95% CI 1.20–1.64) and cardiovascular mortality (HR 1.30; 95% CI 1.08–1.56). [46].

In individuals with MASLD, achieving glycemic control and optimal HbA1c levels requires higher insulin doses compared to T1DM patients without MASLD, who require lower doses to achieve similar diabetes control. [43] This difference can be explained by the unique pathophysiological features of MASLD in T1DM, which include hyperglycemia unrelated to endogenous hyperinsulinemia and/or insulin resistance, secondary to reduced hepatic glucose clearance and insulin resistance in both peripheral tissues and the liver. [47] The requirement for higher insulin doses in MASLD patients with T1DM compared to those without MASLD is clinically observed, but the underlying mechanism involving reduced hepatic glucose clearance remains speculative and is mostly extrapolated from T2DM and insulin resistance models.

Additionally, MASLD is linked to dyslipidemia, characterized by elevated triglycerides and decreased HDL cholesterol levels. These alterations worsen with the progression of MASLD to MASH. Insulin resistance in adipose tissue plays a critical role by increasing lipolysis, which in turn raises circulating free fatty acid levels. These fatty acids are then taken up by the liver, promoting hepatic lipotoxicity and driving the progression of MASLD to MASH. [1].

The bidirectional relationship between T1DM and MASLD amplifies the complications associated with both conditions, profoundly altering their natural course. MASLD is closely tied to hepatic and peripheral insulin resistance, perpetuating a cycle of progressive damage. When these pathologies coexist, the resulting metabolic and cardiovascular complications are exacerbated, underscoring the need to understand their interaction to optimize clinical management and prevent disease progression. The bidirectional nature of the T1DM-MASLD relationship is increasingly supported, as each condition exacerbates the other via overlapping mechanisms of insulin resistance and systemic inflammation, though the full trajectory has been better characterized in T2DM cohorts.

## Molecular implications in T1DM and MASLD

The primary contributors to hepatic triglyceride synthesis include free fatty acids (FFAs) and glycerol. Under physiological conditions, adipose tissue supplies 43.6% of these lipids, de novo lipogenesis accounts for 8.2%, chylomicrons contribute 15.2%, and dietary intake represents 10.3%. [48] However, in patients with MASLD, these proportions are significantly altered. Approximately 59% of hepatic lipids originate from non-esterified circulating fatty acids released through insulin resistance-induced lipolysis in adipose tissue, while 26.1% derive from de novo lipogenesis mediated by SREBP-1c and ChREBP, which are stimulated by hyperinsulinemia and hyperglycemia. Lastly, 14.9% of lipids are obtained directly from dietary sources. Notably, de novo lipogenesis in MASLD patients has been shown to be up to 3.5 times higher than in individuals without MASLD. [49, 50] The redistribution of lipid sources in MASLD—particularly the increased contribution of FFAs from adipose tissue and upregulated de novo lipogenesis—is well-established, primarily supported by human tracer studies and biopsy data in T2DM and MASLD, and likely applicable to T1DM by analogy.

These findings underscore the essential role of adipose tissue insulin resistance in the development and progression of MASLD, as it constitutes the primary source of FFAs for the liver. In addition, lipoproteins play a crucial role in hepatic triglyceride export. One of the key lipoproteins involved is very low-density lipoprotein (VLDL); however, its secretion remains insufficient to counteract the accumulation of hepatic triglycerides, thereby perpetuating hepatic steatosis. This persistent lipid overload exacerbates insulin resistance and contributes to hypertriglyceridemia. Insulin resistance in adipose tissue is a key driver of FFA flux to the liver, and the insufficient secretion of VLDL particles is a recognized contributor to hepatic triglyceride accumulation and hypertriglyceridemia. [51, 52].

The accumulation of intrahepatic lipids triggers the activation of c-Jun N-terminal kinase 1 (JNK1) and protein kinase C epsilon (PKCε), both of which inhibit the phosphorylation of IRS-1 and IRS-2, thereby preventing their activation and promoting insulin resistance. The activation of JNK1 and PKCε in response to lipid overload, and their inhibitory effect on IRS-1/2 phosphorylation, is a well-documented mechanism of insulin resistance in MASLD and T2DM models and plausibly involved in T1DM-associated MASLD. [53].

Furthermore, intracellular diacylglycerol content exhibits a strong correlation with insulin resistance (r = 0.80, p = 0.001), playing a key role in lipid-induced insulin resistance (r = 0.67, p = 0.001). Additionally, diacylglycerol mediates lipid-induced insulin resistance in skeletal muscle through activation of protein kinase C theta (PKCθ). Diacylglycerol accumulation is strongly correlated with hepatic insulin resistance, especially via PKCθ activation in skeletal muscle, supported by human metabolic studies. [54].

These disruptions in intrahepatic lipid metabolism affect multiple pathways, directly impacting the dual phenotype of the disease, namely T1DM and MASLD. These mechanisms likely contribute to the dual phenotype of T1DM with MASLD, although direct mechanistic studies in human T1DM remain limited. Understanding these molecular mechanisms is essential for developing targeted therapeutic strategies to mitigate disease progression and its associated metabolic complications.

### Mitochondrial mechanisms

Mitochondria play a fundamental role in bioenergetic processes, lipid oxidation, intermediate metabolism, apoptosis, mitophagy, and redox homeostasis maintenance. Moreover, they constitute one of the primary sources of oxidative stress involved in the onset and progression of diseases such as hepatic steatosis. [55].

Hepatic mitochondria are also key mediators of metabolic flexibility, which is defined as the ability to adapt to fluctuations in energy demand and supply, thereby ensuring systemic homeostasis. This process involves dynamic adjustments in glucose and free fatty acid oxidation based on substrate availability. In various metabolic disorders such as T2DM, obesity, and MASLD, a reduction in metabolic flexibility has been documented. This impairment is characterized by an increased reliance on free fatty acid oxidation, even when glucose should be the predominant energy source, such as during a euglycemic-hyperinsulinemic clamp. Impaired metabolic flexibility and increased FFA oxidation in MASLD and T2DM have been repeatedly demonstrated in metabolic clamp studies, with emerging but more limited evidence in T1DM. [56, 57].

In patients with T1DM and T2DM, hepatic mitochondrial energy metabolism is impaired, as indicated by reduced levels of adenosine triphosphate (ATP) and inorganic phosphate (Pi). These reductions correlate positively with hepatic insulin sensitivity (r = 0.55, p < 0.05). However, in the specific case of T1DM, changes in ATP and Pi levels occur independently of body weight and hepatic steatosis and are negatively correlated with HbA1c levels (r = −0.56, p < 0.05). In T1DM, decreased hepatic ATP and Pi levels have been observed, correlating inversely with HbA1c and independently of body weight or steatosis, suggesting a distinct mitochondrial phenotype. [58].

When T1DM and MASLD coexist, additional mitochondrial alterations arise, particularly in advanced disease stages. These alterations include megamitochondria with multilamellar membranes, loss of mitochondrial cristae, and the presence of paracrystalline inclusion bodies within mitochondria. Because of increased free fatty acid levels driven by insulin resistance, β-oxidation is upregulated due to mitochondrial morphological changes, leading to excessive production of reactive oxygen species (ROS) and cytotoxic intermediates. The formation of megamitochondria and paracrystalline inclusions in T1DM-MASLD co-occurrence has been reported in advanced stages, though the evidence derives mostly from animal models and histological case series. [59, 60].

In advanced MASLD, mitochondrial respiration deteriorates due to damage to mitochondrial DNA and structural proteins. These alterations, together with hepatic insulin resistance, result in a reduced functional capacity of the electron transport chain, leading to diminished activity of its enzymatic complexes. Progressive mitochondrial dysfunction—particularly affecting the electron transport chain—is a key driver of ROS overproduction and hepatocellular injury in advanced MASLD. [56, 61].

**Fig. 2:**
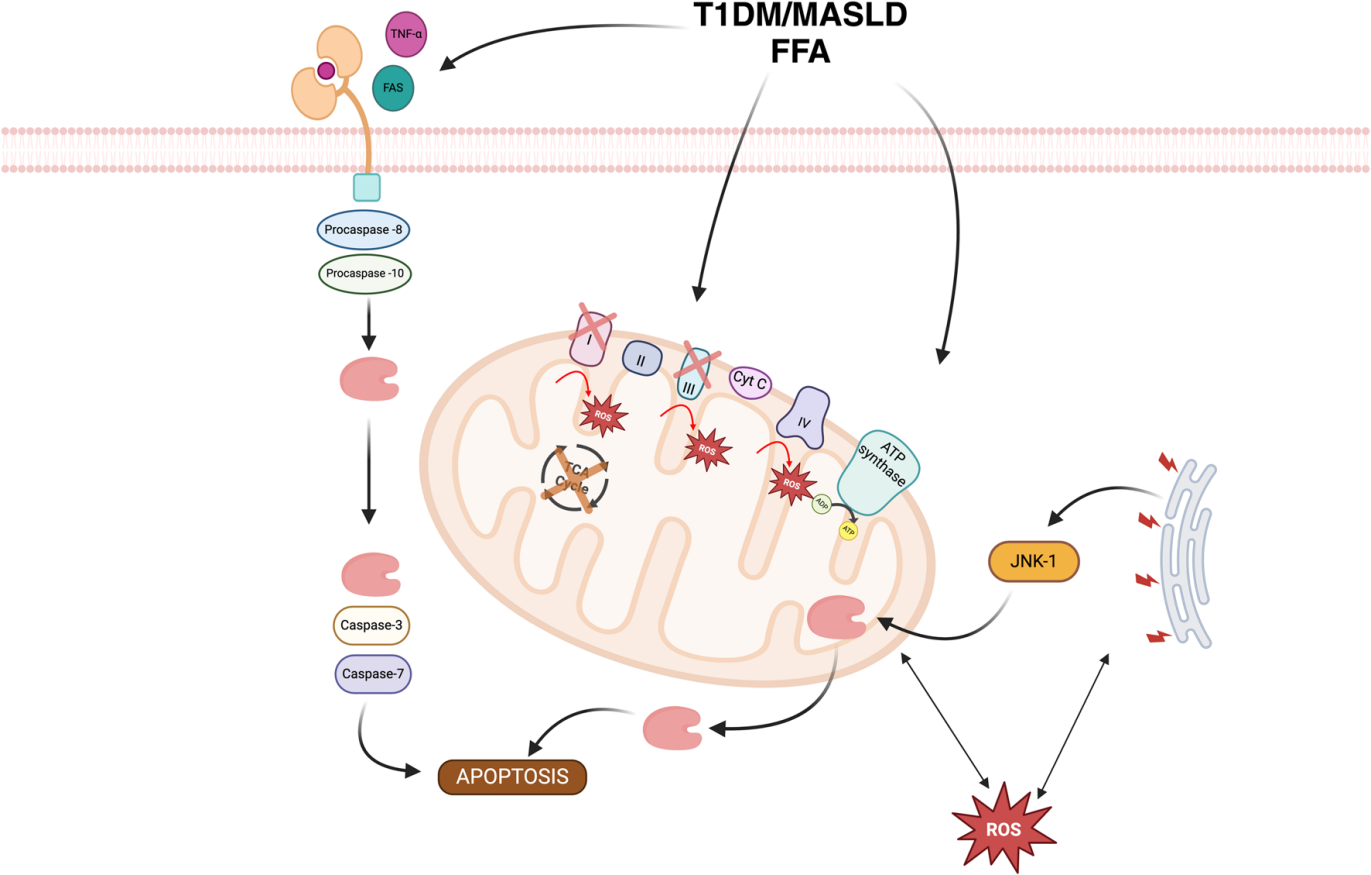
The T1DM/MASLD binomial is a key element in mitochondrial dysfunction, due to increased oxidative stress, inflammation, dysfunctional metabolic pathways, endoplasmic reticulum (ER) stress and activation of cell death pathways

Additionally, mitochondrial dysfunction is accompanied by increased activity of JNK1, a critical regulator in MASLD pathogenesis. Besides the overproduction of ROS, there is a concomitant reduction in hepatic catalase activity, which serves as an essential antioxidant defense mechanism. The decline in catalase function further amplifies oxidative stress, exacerbating mitochondrial damage and disease progression. Hence, the decline in hepatic catalase activity in T1DM patients with MASLD remains speculative, with supportive evidence in animal models. [62] (Fig. 2).

### Inflammatory mechanisms

Adipose tissue macrophages in individuals with obesity exhibit elevated expression of genes encoding pro-inflammatory cytokines, such as tumor necrosis factor-α (TNF-α) and interleukin-6 (IL-6), while concurrently showing reduced expression of interleukin-10 (IL-10), a protective factor against cytokine-induced insulin resistance. Additionally, free fatty acids (FFAs) activate Toll-like receptors 2 and 4 (TLR2 and TLR4), which in turn stimulate the expression of pro-inflammatory genes, activate JNK pathways, and inhibit the nuclear factor kappa-B kinase subunit beta (IKKβ). [63, 64].

TLR4, the first Toll-like receptor to be identified and characterized, is expressed in various hepatic cell types, including hepatocytes, Kupffer cells, and hepatic stellate cells. The TLR4 signaling pathway is closely associated with MASLD pathogenesis and plays a key role in steatosis, hepatitis, and fibrosis. [65].

The activation of IKKβ initiates a phosphorylation, ubiquitination, and subsequent degradation cascade of the inhibitor κB (IκB), thereby allowing nuclear factor kappa-B (NF-κB) to be released and translocated into the nucleus. [66] NF-κB is a critical regulator of inflammation, as its activation and nuclear translocation lead to increased expression of pro-inflammatory cytokines such as TNF-α, IL-6, and IL-1β. These cytokines contribute to both local and systemic inflammatory responses and play an essential role in the development of insulin resistance in the liver and peripheral tissues. [67] The role of TNF-α, IL-6, and IL-1β in promoting hepatic and systemic insulin resistance is robustly supported, across obesity, T2DM, and MASLD models, and increasingly confirmed in T1DM through biomarker studies. TLR4 activation and downstream NF-κB signaling are central in MASLD pathogenesis, though most mechanistic insights derive from animal models and MASLD studies**.**

Since 1993, TNF-α has been recognized as a key factor in insulin resistance, as its concentrations were found to be elevated in the adipose tissue of obese animal models. Neutralizing TNF-α increased peripheral glucose uptake in response to insulin, thereby improving insulin sensitivity. [68] The mechanism by which TNF-α induces insulin resistance involves its interaction with insulin IRS-1 via serine/threonine phosphorylation. This modification inhibits IRS-1 function by blocking insulin receptor autophosphorylation and disrupting downstream signaling. Furthermore, TNF-α has been shown to activate IKKβ and promote IL-6 expression in the liver. [69] The receptor activator of nuclear factor κB ligand (RANKL) is upregulated in individuals with insulin resistance. Its interaction with its receptor, RANK, triggers IKKβ activation, leading to NF-κB activation and promoting the expression of pro-inflammatory cytokines in hepatocytes. [70].

IL-6 plays a pivotal role in insulin resistance by interfering with IRS-1 and insulin receptor interactions in hepatocytes, thereby inhibiting AKT activation and glycogen synthesis. The mechanism by which IL-6 induces insulin resistance involves its intracellular signaling cascade, which includes the activation of Janus kinase (JAK) and signal transducers and activators of transcription 3 and 5B (STAT3/STAT5B). This cascade subsequently induces the expression of suppressor of cytokine signaling 3 (SOCS3), which directly interacts with the insulin receptor β-subunit, inhibiting its autophosphorylation and intracellular signaling. Notably, elevated IL-6 levels in adipose tissue impair insulin-mediated lipolysis, leading to an increased flux of FFAs and glycerol to the liver, exacerbating metabolic dysfunction. [71, 72] The involvement of SOCS3 as a mediator of IL-6–induced insulin resistance is well-characterized, particularly in hepatocytes, and the exact contribution of elevated IL-6 levels in T1DM to hepatic steatosis remains under investigation, although evidence supports its role in impairing lipolysis and enhancing FFA delivery to the liver.

Interleukins from the IL-1 family also play a significant role in insulin resistance due to their strong pro-inflammatory activity. Among them, IL-1β is particularly relevant. IL-1β activation requires caspase-1, which converts its precursor into the active cytokine. This process is linked to inflammasome signaling, which is increasingly recognized as a crucial component of the inflammatory response in MASLD. [73] Furthermore, obesity and high-fat diets have been associated with elevated IL-1β levels. Studies in animal models have demonstrated that the absence of caspase-1 expression reduces IL-1β levels and promotes the differentiation of adipocytes into a lineage more sensitive to insulin. [74].

Moreover, IL-1β directly affects pancreatic β-cells, reducing insulin granule transport to the plasma membrane by approximately 60%, which is linked to a 50% reduction in synaptosome associated protein 25 (SNAP25) protein levels. SNAP25, a member of the SNARE protein family, is essential for insulin granule exocytosis, and its downregulation leads to impaired insulin secretion. The role of IL-1β in impairing SNAP25-mediated insulin granule transport is primarily shown in vitro and in animal models, though it may be pathophysiologically relevant in T1DM. [75, 76].

**Fig. 3:**
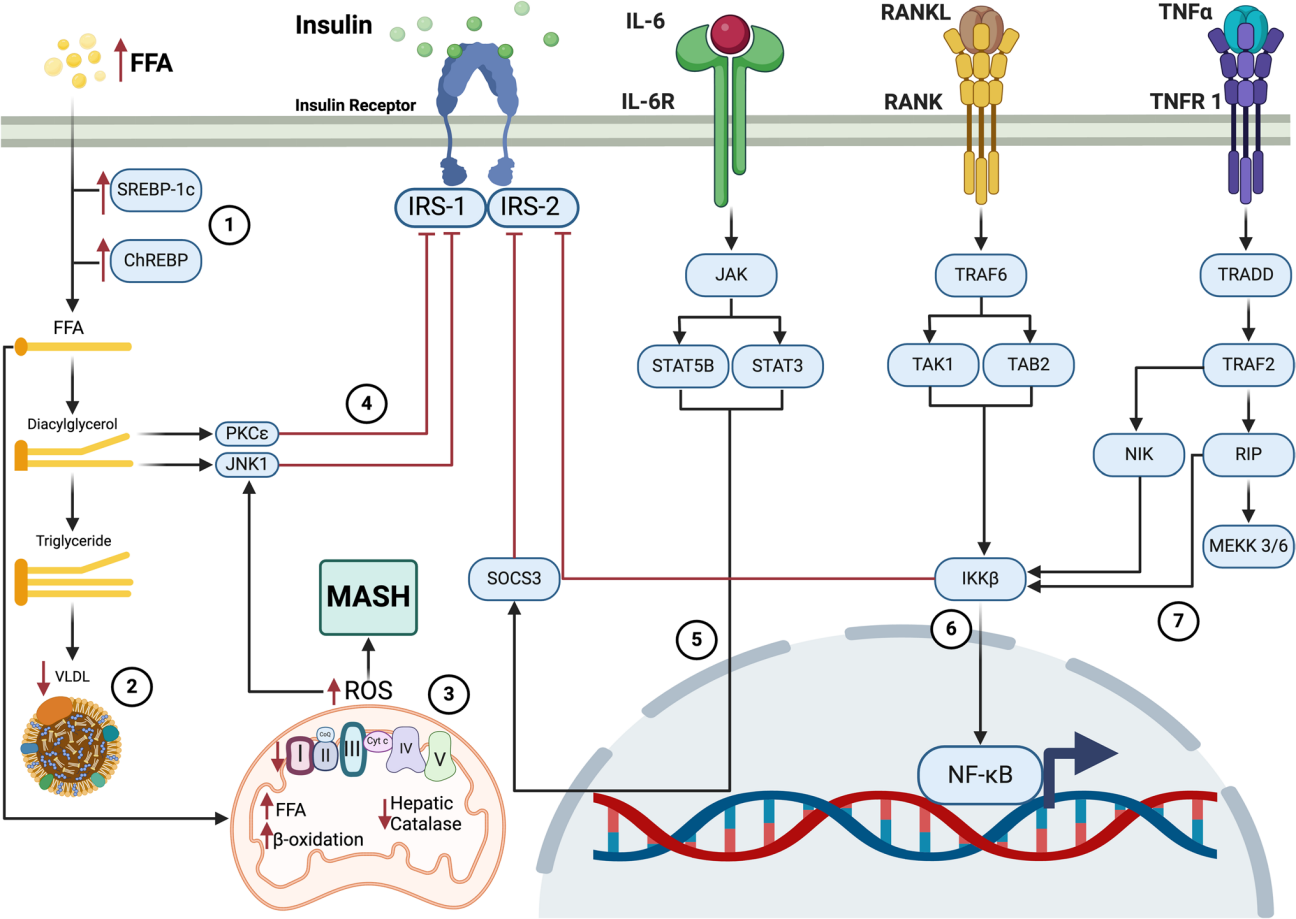
T1DM pathophysiological mechanisms in the liver. 1) An increase in free fatty acids and de novo lipogenesis is observed, so the amount of lipids in the liver will be increased. Liver lipids can be found in 3 forms: free fatty acids, diacylglycerol, and triglycerides. 2) Lipid export by VLDL is not sufficient to compensate for hepatic lipid content. 3) β oxidation is increased due to the large availability of lipids in the liver, hepatic catalase is decreased, and the electron transport chain will also be affected since the action of its complexes is decreased, which increases the ROS and JNK1 production, leading to MASH progression. 4) The increase in lipids leads to the activation of JNK1 and PKCε, which block the phosphorylation of IRS. 5) IL-6 initiates its signaling by activating JAK/STAT that activates SOCS3, which promotes insulin resistance. 6) RANKL via the TRAF6 pathway activates IKKβ, which promotes insulin resistance and activates NF-κB. 7) TNFα, through the activation of the TRAF2 pathway, activates IKKβ inducing NF-κB, which increases the expression of proinflammatory cytokines. **Acronyms: ChREBP**: Carbohydrate response element binding-protein, **FFA**: Free fatty acid, **IKKβ**: Inhibitor of NF-κB kinase β, **IL-6**: Interleukin-6, **IL-6R**: Interleukin-6 receptor, **IRS-1**: Insulin receptor substrate 1, **IRS-2**: Insulin receptor substrate 2, **JAK**: Janus kinase, **JNK1**: c-Jun N-terminal kinase 1, **MEKK 3/6**: Mitogen-activated protein kinase kinase 3/6, **MASH**: Metabolic dysfunction-associated steatohepatitis, **NF-κB**: Nuclear factor kappa B, **NIK**: NF-κB-inducing kinase, **PKCε**: Protein kinase C-epsilon, **RANK**: Receptor activator of nuclear factor κ B, **RANKL**: Receptor activator for nuclear factor κ B ligand, **RIP**: Receptor-interacting protein, **ROS**: Reactive oxygen species, **SOCS3**: Supressor of cytokine signaling 3, **SREBP-1c**: Sterol regulatory element binding-protein 1c, **STAT3**: Signal transducer and activator of transcription 3, **STAT5B**: Signal transducer and activator of transcription 5B, TAB2: TGFβ-activated kinase 1 and MAP3K-binding protein 2, **TAK1**: Mitogen-activated protein kinase kinase kinase, **TNF α**: Tumor necrosis factor α, **TNFR 1**: Tumor necrosis factor receptor 1, **TRADD**: Tumor necrosis factor receptor type 1-associated DEATH domain protein, **TRAF2**: TNF receptor associated factor 2, **TRAF6**: TNF receptor associated factor 6, **VLDL**: Very low density lipoprotein

Conversely, certain interleukins within the IL-1 family exert opposing effects to IL-1β. For instance, IL-18 exhibits hypoglycemic properties by inhibiting the expression of gluconeogenic genes such as phosphoenolpyruvate carboxykinase 1 (PCK1) in the liver. [77] Similarly, IL-37 has potent anti-inflammatory effects mediated through Smad3, which acts as an antagonist of STAT3, thereby attenuating dendritic cell activation and limiting the pro-inflammatory response. [78] The protective roles of IL-18 and IL-37 are emerging and primarily demonstrated in preclinical settings, with limited translational data in human T1DM or MASLD. (Fig. 3).

## Potential treatments

The most effective treatment for MASLD is lifestyle modification, including diet and exercise. A weight loss of at least 7% of baseline body weight has been shown to improve insulin resistance, reduce blood pressure, lower cardiovascular risk, and decrease pro-inflammatory cytokines. [79].

### Established and routinely used therapies

A study involving patients with obesity and MASLD who underwent a 48-week treatment with metformin demonstrated that 30% experienced weight reduction and histological improvement. [80].

On the other hand, thiazolidinediones have been shown to enhance insulin sensitivity. The use of pioglitazone has been tested in MASH, yielding beneficial results. In an 18-month study using a 45 mg/day dose, 58% of patients showed a reduction of at least two points in two categories of the Kleiner score without an increase in fibrosis level. Furthermore, 51% of patients achieved MASH resolution, and hepatic steatosis decreased from 19 to 7% by the end of the study. These effects persisted for 36 months after treatment cessation. Additionally, pioglitazone may reduce the visceral-to-subcutaneous fat ratio, leading to increased plasma adiponectin levels. These changes likely underlie the reduction in steatosis and necroinflammation observed in MASLD patients. [60, 81].

Exenatide, a glucose-lowering agent belonging to the glucagon-like peptide-1 receptor agonists (GLP-1 RA), promotes weight loss, reduces insulin requirements, and enhances insulin sensitivity. In a 12-month prospective trial, the effects of oral and subcutaneous semaglutide were evaluated, showing not only its weight-loss capabilities but also histological improvements in MASLD patients. [82, 83].

Empagliflozin, a sodium-glucose cotransporter-2 (SGLT2) inhibitor, has demonstrated an 8% reduction in baseline HbA1c levels, an average weight loss of 2.6 kg, and a decrease in basal insulin requirements from 25.7 IU/day to 19.5 IU/day. [20].

Other drugs not directly related to diabetes have been explored for T1DM and MASLD.

### Emerging or investigational therapies

Anakinra, an interleukin-1α and IL-1β inhibitor that acts through competitive receptor binding, has demonstrated beneficial effects, including a 0.46% reduction in HbA1c levels (p = 0.004, 95% CI 0.11–0.60) and decreased IL-6 expression. However, it did not improve insulin sensitivity. [84].

The molecule RO5093151, a potent and selective inhibitor of 11β-hydroxysteroid dehydrogenase type 1 (11β-HSD1), regulates hepatic glucose production and lipid deposition. Patients treated with RO5093151 for 12 weeks exhibited a 2.57% reduction in hepatic steatosis (p = 0.02), accompanied by an average weight loss of 1.98 kg and a decrease in total, visceral, and subcutaneous adipose tissue. Additionally, 20% of treated patients achieved MASLD resolution. Adverse effects included gastrointestinal disorders, infections, and a higher prevalence of neurological disturbances. [85].

A novel pharmacological approach for MASH treatment is obeticholic acid, which has shown both histological and biochemical improvements. In 38% of patients, fibrosis decreased by at least one stage; however, only 28% experienced no worsening of MASH (RR = 2.2, 95% CI 1.4–3.2, p = 0.0001). Additionally, 91% of patients reported adverse effects such as pruritus and increased LDL levels accompanied by decreased HDL levels. [86].

Lanifibranor, a peroxisome proliferator-activated receptor (PPAR) agonist, has shown promising results in MASLD treatment. In the phase 2b NATIVE clinical trial, which included 247 patients with"high-risk MASH,"lanifibranor significantly improved MASH resolution without worsening fibrosis at week 24. This benefit was observed in 49% and 39% of patients receiving 1200 mg and 800 mg of lanifibranor, respectively, compared to 22% in the placebo group. Moreover, lanifibranor demonstrated positive effects on insulin resistance, as assessed by the homeostatic model assessment of insulin resistance (HOMA-IR), and on glycemic control, reinforcing its potential for managing the MASLD-T1DM comorbidity. [87, 88].

Antioxidant therapy has also been explored. One of the most studied antioxidants is vitamin E, which has been shown to improve hepatic steatosis after 96 weeks of treatment with 800 IU daily. Improvements were observed in 43% of patients (p = 0.001), with at least a two-point reduction in the Kleiner score without fibrosis progression. Additionally, biochemical parameters improved, with decreased ALT and AST levels. [89] Despite its benefits, the European Association for the Study of the Liver (EASL) guidelines caution against its widespread use due to its association with increased mortality from hemorrhagic disease and prostate cancer. [90, 91].

In conclusion, managing MASLD-T1DM requires a personalized, multifaceted approach that includes lifestyle modifications and targeted therapies. Although several agents have shown promising efficacy, further studies are needed to validate their long-term safety and applicability in this specific patient population. The proposed treatments for MASLD-T1DM are summarized in Table 3.

**Table 3:** Possible treatments for the T1DM-MASLD binomial

Drug	Drug family	Advantage	References
Metformin	Biguanides	Weight loss, increased glomerular filtration rate, decreased LDL levels and MASH activity	[81]
Pioglitazone	Thiazolidinediones	Increased insulin sensitivity and decreased MASH activity	[61, 82]
Exenatide	GLP-1	Weight loss, decreased insulin requirements and increased insulin sensitivity	[83, 84]
Empagliflozin	SGLT2	Weight loss, decreased insulin requirements and glycated hemoglobin	[20]
Anakinra	Interleukin inhibitor	Decreased glycated hemoglobin, fasting plasma glucose, IL-6 and C-reactive protein levels	[85]
RO5093151	Selective inhibitor of 11β-HSD1	Weight loss, decreased hepatic steatosis and total body fat	[86]
Obeticholic acid	Selective farnesoid X receptor agonist	Decreased fibrosis and reduction of hepatic transaminases	[87]
Lanifibranor	PPAR agonist	Decreased MASH activity	[88]
Vitamin E	Antioxidant	Decreased MASH activity and reduction of hepatic transaminases	[90]

## Discussion

Evidence suggests that the traditional view of insulin resistance as exclusive to T2DM is no longer accurate, as it also occurs in a substantial subset of individuals with T1DM. The coexistence of MASLD in patients with T1DM is associated with a markedly increased risk of both microvascular and macrovascular complications, including MASH, chronic kidney disease, atherosclerosis, and increased cardiometabolic morbidity and mortality—positioning this overlap as an emerging public health concern.

The interplay between T1DM and MASLD presents unique pathophysiological, diagnostic, and therapeutic challenges. Although important progress has been made in the development of pharmacological therapies for MASLD, most clinical trials have focused on T2DM or general MASLD populations, with limited inclusion of T1DM cohorts. This results in a knowledge gap and therapeutic uncertainty for a growing patient population with distinct metabolic characteristics.

Looking forward, several research priorities should be emphasized to advance the understanding and management of MASLD in T1DM. These include the implementation of prospective cohort studies using standardized liver imaging techniques to accurately determine MASLD prevalence and track disease progression in T1DM populations. Additionally, there is a pressing need to validate MASLD-specific risk stratification tools that account for the unique metabolic features of T1DM, such as exogenous insulin administration and altered portal-systemic insulin gradients. Clinical trials should be designed to assess the safety and efficacy of antifibrotic, insulin-sensitizing, and anti-inflammatory therapies directly in T1DM patients with MASLD, rather than relying on extrapolated data from T2DM studies. Finally, deeper investigation into the molecular and immunometabolic mechanisms that characterize the T1DM–MASLD overlap will be essential for developing precision medicine strategies tailored to this distinct patient population.

## Conclusion

Addressing this comorbidity requires a comprehensive, multidisciplinary strategy that includes early screening, personalized treatment plans, and the design of targeted clinical trials. Strengthening the evidence base for MASLD management in T1DM will be essential to improving outcomes and reducing long-term complications in this vulnerable patient population.

## Data Availability

The data are available upon request from the corresponding author.
